# SMI of Bcl-2 TW-37 is active across a spectrum of B-cell tumors irrespective of their proliferative and differentiation status

**DOI:** 10.1186/1756-8722-2-8

**Published:** 2009-02-16

**Authors:** Ayad M Al-Katib, Yuan Sun, Anton Scott Goustin, Asfar Sohail Azmi, Ben Chen, Amro Aboukameel, Ramzi M Mohammad

**Affiliations:** 1 Department of Internal Medicine, Division of Hematology/Oncology, Wayne State University School of Medicine, Detroit, Michigan, USA

## Abstract

The Bcl-2 family of proteins is critical to the life and death of malignant B-lymphocytes. Interfering with their activity using small-molecule inhibitors (SMI) is being explored as a new therapeutic strategy for treating B-cell tumors. We evaluated the efficacy of TW-37, a non-peptidic SMI of Bcl-2 against a range spectrum of human B-cell lines, fresh patient samples and animal xenograft models. Multiple cytochemical and molecular approaches such as acridine orange/ethidium bromide assay for apoptosis, co-immunoprecipitation of complexes and western blot analysis, caspase luminescent activity assay and apoptotic DNA fragmentation assay were used to demonstrate the effect of TW-37 on different B-cell lines, patient derived samples, as well as in animal xenograft models. Nanomolar concentrations of TW-37 were able to induce apoptosis in both fresh samples and established cell lines with IC_50 _in most cases of 165–320 nM. Apoptosis was independent of proliferative status or pathological classification of B-cell tumor. TW-37 was able to block Bim-Bcl-X_L _and Bim-Mcl-1 heterodimerization and induced apoptosis via activation of caspases -9, -3, PARP and DNA fragmentation. TW-37 administered to tumor-bearing SCID mice led to significant tumor growth inhibition (T/C), tumor growth delay (T-C) and Log_10_kill, when used at its maximum tolerated dose (40 mg/kg × 3 days) via tail vein. TW-37 failed to induce changes in the Bcl-2 proteins levels suggesting that assessment of baseline Bcl-2 family proteins can be used to predict response to the drug. These findings indicate activity of TW-37 across the spectrum of human B-cell tumors and support the concept of targeting the Bcl-2 system as a therapeutic strategy regardless of the stage of B-cell differentiation.

## Background

Lymphoid cancers are common in the US. They include a heterogeneous group of diseases spanning the full spectrum of both T- and B- cell differentiation stages. Non-Hodgkin's lymphoma (NHL), the most common among these disorders, is the 5^th ^and 6^th ^most common cancer among the male and female US population, respectively [1]. When combined with other lymphoid cancers like multiple myeloma (MM), acute lymphoblastic leukemia (ALL) and chronic lymphocytic leukemia (CLL), these diseases form more than 7% of all cancers in the US with more than 103,000 cases estimated to be diagnosed in 2007 [1].

There are different ways of classifying malignant lymphoid disorders based on morphology, clinical behavior, cell lineage, immunophenotypes, genetic abnormalities or a combination of these features [2-4]. We have chosen to catalogue malignant B-lymphoid disorders according to the state of differentiation they represent and established a number of cell lines representing them [5]. According to this schema, B-cell tumors are believed to represent discrete stages of B-cell differentiation from the most immature (like ALL) to the most mature (like MM and Waldenstrom's Macroglobulinemia [WM]) stages. Disorders of the early stages (ALL, high grade NHL) are curable with chemotherapy that is the mainstay of treatment, whereas tumors of the more mature stages (like low grade NHL, CLL, WM, MM) remain incurable [6]. At the molecular genetic level, most of these disorders are characterized by very well defined, specific non-random abnormalities that are potential targets for new therapy. Among the most common molecular genetic abnormalities in lymphoid tumors are those involving Bcl-2 and other apoptosis-regulating molecules [7-9].

Recent research efforts have yielded a number of synthetic small molecules capable of interfering with cellular pathways [10-13]. One such small molecule inhibitor (SMI) is TW-37 [14]. This compound binds with high affinity to the hydrophobic groove found in the multidomain anti-apoptotic Bcl-2 family proteins; this groove is naturally the site for interaction with BH3 alpha helix in the BH3-only pro-apoptotic proteins. Drug binding is thought to block the anti-apoptotic proteins from heterodimerizing with the pro-apoptotic members of the Bcl-2 family (Bad, Bid, Bim) or may produce conformational changes that disable the anti-apoptotic members. It is well known that over expression of anti-apoptotic Bcl-2 proteins leads to apoptosis-resistance and is believed to be a major reason for treatment failure in lymphoid tumors [15-19]. In this report, we show that exposure of a variety of B-cell tumor cells to TW-37 is sufficient to inhibit growth and induce apoptosis. The study mechanistically demonstrates the clinical relevance of the Bcl-2 system as therapeutic target in these tumors.

## Materials and methods

### TW-37

Design, synthesis, purification, and chemical characterization of TW-37 N-[(2-tert-butyl-benzenesulfonyl)-phenyl]-2,3,4-trihydroxy-5-(2-isopropyl-benzyl)-benzamide is described in detail in ref [14]; in the inactive congener TW-37a, all three hydroxyl groups in the polyphenolic ring have been substituted with a methyl group, resulting in a 100-fold loss of binding.

### Cell lines and patient-derived primary lymphocytes

The acute lymphoblastic leukemia (WSU-pre-B-ALL), diffuse large cell lymphoma cell line (WSU-DLCL_2_), follicular small cleaved cell lymphoma (WSU-FSCCL) and Waldenstrom's macroglobulinemia (WSU-WM) cell lines were established in our laboratory at the Wayne State University School of Medicine [20-23]. The WSU-pre-B-ALL cell line is CD10+, CD19+, CD20+, TdT+; the WSU-DLCL2 and WSU-FSCCL are both mature (SIg+), CD20+ cell lines. The WSU-WM cell line is IgM-secreting cell line. Fresh peripheral blood samples were obtained from patients with active chronic lymphocytic leukemia (CLL)/small lymphocytic lymphoma (SLL) or marginal zone lymphoma (MZL) in leukemic phase under IRB-approved protocol and used to assess the TW-37 cytotoxic effect on primary lymphoma cells. The CLL/SLL cells expressed CD5, CD19, CD20 and faint monotypic SIg. The MZL cells were CD5-, CD19+ and CD20+. Mononuclear cells were separated by Ficoll-Hypaque density centrifugation (Lymphoprep™, Fresenius Kabi Norge AS, Oslo, Norway), washed twice with PBS and then cell pellet was resuspended in RPMI-1640 culture medium.

### Effect of TW-37 on Growth of established cell lines and fresh lymphoma cells

Cells from established lines (above) were plated in 24-well culture clusters (Costar, Cambridge, MA) at a density of 2 × 10^5 ^viable cells/ml/well. Triplicate wells were treated with 0.0–750 nM TW-37. Plates were incubated at 37°C in a humidified incubator with 5% CO_2_. All cultures were monitored throughout the experiment by cell count and viability every 24 hr for 72 hr using 0.4% trypan blue stain (Gibco BRL, Grand Island, NY) and a hemacytometer. Fresh primary lymphoma cells isolated from patients were processed similarly except cells were seeded at a density of 5 × 10^5^/ml/well. Statistical analysis was performed using the *t *test, two-tailed, with 95% confidence intervals between treated and untreated samples. *P *value < 0.05 were used to indicate statistical significance.

### Acridine orange/ethidium bromide (AO/EB) assay for apoptosis

After exposure to various concentrations of TW-37 for 48 or 72 hr, cells were collected by centrifugation and resuspended into 25 μl of PBS. One microliter of AO/EB mix was added to each sample prior to analysis by fluorescent microscope. Using fluorescence microscope, cells seen in orange or light orange were counted as apoptotic whereas cells in green or light green were counted as viable [24]. Data analysis was done using "GraphPad Prism 4.03" software.

### Bcl-2 family protein expression profiling, caspase and PARP cleavage assays by Western blots

Bcl-2 family protein expression profile without TW-37 treatment among 4 WSU lymphoma cell lines was determined as baseline as previously described [25]. Cells were seeded and cultured in T-75 cell culture flasks and harvested at exponential growth phase. Cells were lysed by buffer containing 50 mM Tris-HCL, 1% NP-40, 0.1% SDS, 150 mM NaCl, 1 mM EDTA, 1 mM PMSF, 1 mM Na_3_VO_4 _and protease inhibitor and total protein quantification determined using Protein Assay (BioRad, Hercules, CA). For Western Blotting, 40 or 100 μg of total protein was separated by 12% or 15% SDS-PAGE gel electrophoresis then transferred to nitrocellulose membrane (BioRad, Hercules, CA). Membranes were blocked with 5% Fat Free Dry Milk and subjected to immunoblotting using antibodies against individual human Bcl-2 family proteins [Pro-Apoptosis Bcl-2 Family Antibody Sampler Kit or Pro-Survival Bcl-2 Family Antibody Sampler Kit (Cell Signaling Technology, Beverly, MA)] at 4°C overnight with agitation. After 3 washings, of 15 min each, membranes were blotted with horseradish peroxidase HRP-conjugated secondary antibody at room temperature for 2 hr. Following 3 washings of each membrane, protein was detected by ECL Western blotting detect reagent (GE Healthcare, Piscataway, NJ). Fresh patient samples were analyzed by the same method. All membranes in each experiment were stripped, blocked and further immunoblotted with anti-*β*-actin (Santa Cruz, Santa Cruz, CA) antibody to confirm equal loading and as reference for quantification of Bcl-2 family protein expression level among each cell line and sample. Expression level of each Bcl-2 family protein was determined by scanning band density using "AlphaEaseFC" software and normalized to density of the *β*-actin band of same sample and the quantification of the Bcl-2 family protein inventory, relative to *β*-actin, was tabulated. Similar procedures were used for TW-37 or TW-37a-treated cells and to detect caspase 3, 8, 9 and PARP cleavage using appropriate antibodies (Cell Signaling Technology, Beverly, MA).

### Caspase luminescent activity assay

Cells were seeded on white Luminometer 96-well plate (Fisher Scientific, Hanover Park, IL) at 2 × 10^4 ^cells per 100 μl/well with various concentrations of TW-37 or 300 nM of TW-37a and cultured at 37°C, 5% CO_2_. Caspase activity assay was performed after 4, 8, 2 and 24 hr of treatment using Caspase-Glo3/7 Assay and Caspase-Glo 9 Assay kit (Promega, Madison, WI). Assay procedure was done following manufacture's instruction using culture media without cells as blank control. One hundred μl of pre-mixed Caspase-Glo mixture was added to each assaying well with shake at 300 rpm for 30 seconds then incubated at room temperature protected from light for 1 to 3 hr. Luminescence was measured by Tecan Multifunction microplate reader at OD_450 _nm versus OD_595 _nm. Data was normalized by substituting substrate with blank control and analyzed by "GraphPad Prism 4.03" software. Statistical analysis was done using two-tailed t-test.

### Apoptotic DNA fragmentation assay

WSU-DLCL_2 _and WSU-FSCCL cells were exposed to TW-37 or its trimethylated enantiomer (TW-37a) for 24 and 48 hr. 4 × 10^6 ^cells were harvested from each condition and subsequently analyzed for DNA fragmentation using Apoptotic DNA Ladder Kit (Roche, Indianapolis, IN). DNA extraction procedure was done following manufacturer's instruction. DNA ladder was visualized by UV spectrometer after 1% agarose gel electrophoresis.

### Co-immunoprecipitation of complexes and Western blot analysis

WSU-FSCCL cells were exposed to 1 or 2 μM TW-37 or TW-37-A for 24 hr then lysed in buffer containing 50 mM Tris·HCL, 1% CHAPS, 0.1% SDS, 150 mM NaCL, 1 mM EDTA, 1 mM PMSF, 1 mM Na_3_VO_4 _and protease inhibitor. 300 μg of total protein from each lysate was subjected for immunoprecipitation anti-Bim (Calbiochem, Darmstadt, Germany) in a total volume of 200 μl at 4°C with agitation. Supernatant was detected by Western blot with anti-Bim, anti-BclX_L _(Calbiochem, Darmstadt, Germany) or anti-Mcl-1 (BD Biosciences, San Diego, CA) antibody and further detected with anti-Actin antibody (Santa Cruz, Santa Cruz, CA).

### SCID-mouse xenografts

Four-week-old female ICR-SCID mice were obtained from Taconic Laboratory (Germantown, NY). The mice were adapted for several days and WSU-DLCL_2 _xenografts were developed as described previously [26]. Each mouse received 10^7 ^WSU-DLCL_2 _cells (in serum-free RPMI-1640) subcutaneously (SC) in each flank area. When SC tumors developed to approximately 1500 mg, mice were euthanized, tumors dissected and mechanically dissociated into single-cell suspensions. Mononuclear cells were separated by Ficoll-Hypaque density centrifugation and washed twice with RPMI-1640 medium. These cells were subjected to phenotypic analysis for comparison with the established tumor cell line to insure the human origin and its stability. After formation of SC tumors, serial propagation was accomplished by excising the tumors, trimming extraneous materials, cutting the tumors into fragments of 20 to 30 mg that are transplanted SC using a 12 gauge trocar into the flanks of a new group of mice.

### Efficacy trial design for TW-37

The maximum tolerated dose (MTD) for TW-37 is defined as the dose that will lead to no deaths of any of the animals and no more than 10% loss of body weight during treatment, followed by weight gain. To test the efficacy of TW-37 *in vivo*, small fragments of WSU-DLCL_2 _xenograft were implanted SC bilaterally into naïve SCID mice as previously described. Mice were checked three times per week for tumor development. Once transplanted WSU-DLCL_2 _fragments developed into palpable tumors (60–100 mg), groups of five animals were removed randomly and assigned to receive TW-37 or diluent (as control). Mice were observed for measurement of SC tumors, changes in weight and side effects of the drug. SC tumors were measured three times per week.

### Assessment of Tumor Response

The end-points for assessing anti-tumor activity were according to standard procedures used in our laboratory and are as follows: Tumor weight (mg) = (A × B^2^)/2, where A and B are the tumor length and width (in mm), respectively; Tumor growth inhibition (T/C) is calculated by using the median tumor weight in the treated group (T) when the median tumor weight in the control group (C) reached approximately 900 mg. Tumor growth delay (T-C) is the difference between the median time (in days) required for the treatment group tumors (T) to reach 900 mg and the median time (in days) for the control group tumors (C) to reach the same weight and tumor cell kill (log_10_) total = (T-C)-/(3.32)(Td).

All studies involving mice were performed under Animal Investigation Committee (AIC)-approved protocols. Tumor weights in SCID mice were plotted against time on a semi-log sheet with the growth pattern resembling an S-shape. Tumor doubling (Td) is the time (in days) required in order for the tumor to double its weight during the exponential growth phase.

### Statistical analysis

For the comparison of tumor weight, the power to detect differences in the mean tumor weight at the completion of treatment between treatment and control groups has been calculated based upon a sample of 5 mice/10 xenografted tumors per group. Power calculations assume that the use of a two-sided, two-sample, *t*-test, with equal variance, and assuming the difference between means to be a proportion of the standard deviation of the outcome measurement. For example, a 1-unit difference between groups represents a difference of one standard deviation between groups. The study has at least 90% power to detect differences larger than 1.6 units of standard deviation between groups.

## Results

### Effect of TW-37 on growth of established malignant lymphoid cell lines and patient-derived lymphoma cells

**Figure 1:**
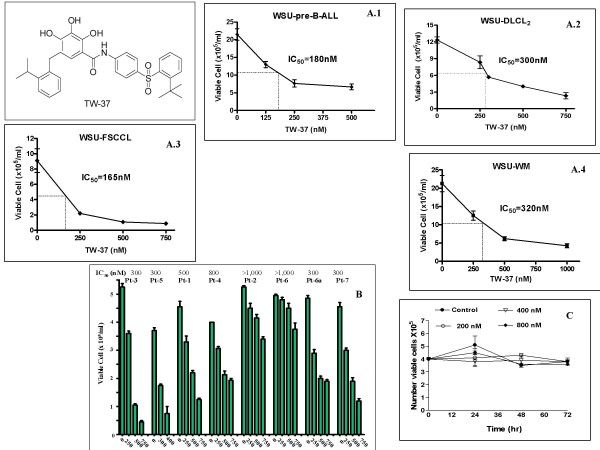
**Structure of small molecule inhibitor TW-37**. Growth inhibition effect of TW-37 on 4 NHL cell lines and fresh cells obtained from 8 patient samples. Data represent IC_50 _at 72 hr from TW-37 exposure using trypan blue exclusion method. **A) **WSU-pre-ALL cell line (A1); WSU-DLCL2 cell line (A2); WSU-FSCCL cell line (A3) and WSU-WM cell line (A4). Cell lines were seeded in 24-well culture clusters at a density of 2 × 10^5 ^viable cells/ml per well. Triplicate wells were treated with 0.0–750 nM TW-37 and incubated for up to 72 hr. **B) **Fresh patient derived cells were seeded in 24-well culture clusters at a density of 5 × 10^5 ^viable cells/ml per well. Triplicate wells were treated with 0.00–750 nM TW-37 and incubated for 72 hr. Cytotoxic effect of TW-37 on primary NHL cells is at 72 hr. **C) **Cytotoxic effect of TW-37 on normal peripheral blood lymphocytes was assayed by seeding 4 × 10^5 ^viable cell/ml and treated with 0.00–800 nM of TW-37 for up to 72 hr.

The structure of TW-37 is given in Figure 1. The cell lines selected span the spectrum of the B-cell lineage. In addition, fresh peripheral blood samples of patients with CLL or leukemic phase of NHL were obtained under IRB-approved protocol. In each case, cells were exposed to TW-37 and TW-37a over 72 hr, and cell viability was determined. In general, exposure to TW-37 resulted in a dose-dependant inhibition of cell proliferation. The TW-37 concentration resulting in 50% growth inhibition (IC_50_) of the established cell lines (Fig. 1A) were as follows: WSU-pre-B-ALL 180 nM (A.1); WSU-DLCL_2 _300 nM (A.2); WSU-FSCCL 165 nM (A.3); WSU-WM 320 nM (A.4). We have similarly tested growth inhibitory effect of TW-37 on 8 patient samples (pt) obtained from 7 patients. Patients 1–6 have a diagnosis of CLL/SLL whereas patient-7 has a diagnosis of marginal zone lymphoma (MZL). Two samples were obtained from case #6; one before therapy (pt.6a), and the second (pt.6) while the patient was on therapy with Rituximab and prednisone. None of the other patients were under active therapy at the time of obtaining blood samples except pt.2 who was receiving pulse dose chlorambucil and prednisone. There was no significant increase in cell numbers of control cultures after 72 hr; however, TW37-treated cultures showed progressive decrease in cell numbers, which was dose dependent (Fig. 1B). This was true of all patient samples although the effect was less profound in cells from pt.2 and pt.6 who were under treatment with chemotherapy for CLL/SLL. The inactive congener TW-37a had no effect (data not shown). Moreover, TW-37 had no effect on normal PBL (Fig. 1C).

### TW-37 activates the caspase pathway and induces apoptosis

Since TW-37 targets proteins in the apoptotic pathway; we investigated its ability to induce apoptotic cell death in lymphoid cell lines and patients samples:

### Apoptosis

**Figure 2:**
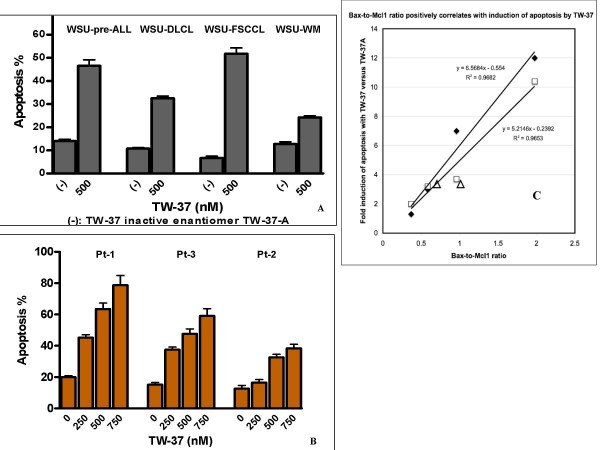
**Acridine orange/ethidium bromide (AO/EB) staining showing apoptosis induction by TW-37**. A, Apoptosis induction of TW-37 on 4 WSU-cell lines was assayed after 72-h exposure. WSU cell lines were seeded and treated with 500 nM of TW-37 and with inactive congener TW-37a [designated as "(-)"] in triplicate and apoptosis was determined by AO/EB staining after 72 h. B, Apoptosis induction of TW-37 on patient-derived NHL cells was determined on 3 selected samples. Apoptotic cells were assayed by AO/EB staining after exposure of TW-37 with concentrations ranging from 0 to 750 nM (0 is the same as TW-37a). C, Bax-to-Mcl-1 ratio positively correlates with induction of apoptosis by TW-37. The Bax/Mcl-1 ratio was plotted on the abscissa against this AO/EB metric on the ordinate for four cell lines (the filled diamonds represent 48 h and the empty squares represent 72 h treatments). Each line is calculated by linear regression using equal weighting of the four points; the lines described closely emanate from the origin (x-intercept = 0.046 to 0.084). Patient data (Patients-1 and 3, empty triangles) lie close to the lines fitted to the data for the four established NHL cell lines.

TW-37 induced significant apoptosis in the cell lines and fresh patient samples (Fig. 2). This effect was specific since there was significant difference between TW-37 and TW-37a used under the same conditions. The highest proportion of cells in apoptosis was observed in WSU-FSCCL indicating higher sensitivity to TW-37 whereas the lowest was in WSU-WM (Fig. 2A). Similarly, TW-37 induced apoptosis on each of the three patient samples examined (Fig. 2B) with lower values in pt.2 that also showed less growth inhibition (Fig. 1B). Interestingly, the Bax-to-Mcl-1 ratio positively correlated with induction of apoptosis in the cell lines and in the 2 fresh cases studied (R^2 ^= 0.9682 and 0.9653 after 48 and 72 h of exposure to TW-37, respectively, Figure 2C).

### Caspase activation, PARP cleavage and DNA fragmentation

**Figure 3:**
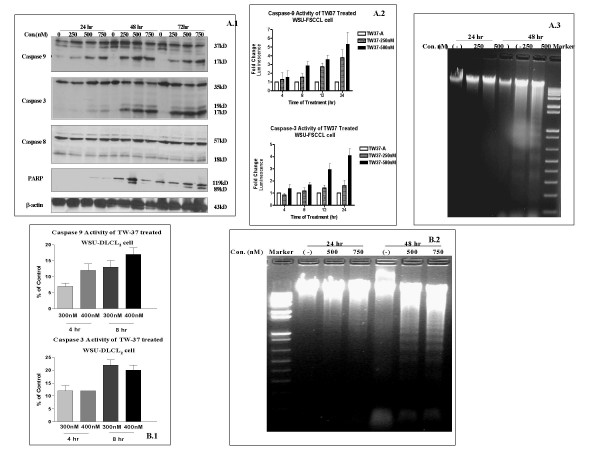
**Cleavage of caspase 9, 3 and PARP protein and induction of Caspase 3, 9 activity and resulting DNA fragmentation in TW-37 treated lymphoid cell lines**. **A) **WSU-FSCCL cells were exposed to TW-37 (0 to 750 nM) at 24, 48 and 72 hr. Caspase 3, 9, 8 and PARP protein cleavage was detected by Western blot **(3A.1)**. Or cells were treated with TW-37 at 0, 250 and 500 nM on white 96-well plate for 4, 8, 12 and 24 hr. Caspase 3, 9 activities were determined by Luminescence immediately on 96-well plate **(3A.2)**. WSU-FSCCL cells treated with TW-37 at 250 and 500 nM. DNA fragmentation was seen evident after 48 hr **(3A.3)**. **(3B1.) **Caspase 9 and 3 enzyme activation by TW-37 was also determined using WSU-DLCL2 lymphoid cell line. Cells exposed to 300, 400 nM of TW-37 for 4 or 8 hr, caspase 9 and 3 activity was detected. **(3B.2) **WSU-DLCL2 cells treated with TW-37 at 500 and 750 nM, DNA fragmentation was evident after 48 hr.

Exposure of WSU-FSCCL cells to TW-37 induced activation of caspase 9 and caspase 3 activity and PARP cleavage (Fig. 3A.1). Using luminescent assay, Caspase activation was evident within 24 hr (Fig. 3A.2) and became more pronounced with longer incubation. Caspase 3 and 9 activation was evident as early as 4 hr after exposure to TW-37, which was again specific to TW-37. There was no activation of caspase 8. TW-37 also induced caspase 3 and 9 activation on WSU-DLCL_2 _cells (Fig. 3B.1). To confirm induction of apoptosis, there was clear evidence of DNA fragmentation of extracts from both WSU-FSCCL and WSU-DLCL_2 _cells (Fig. 3A.3 and 3B.2).

### Baseline expression of Bcl-2 family proteins in cell lines and fresh lymphoma cases

**Figure 4:**
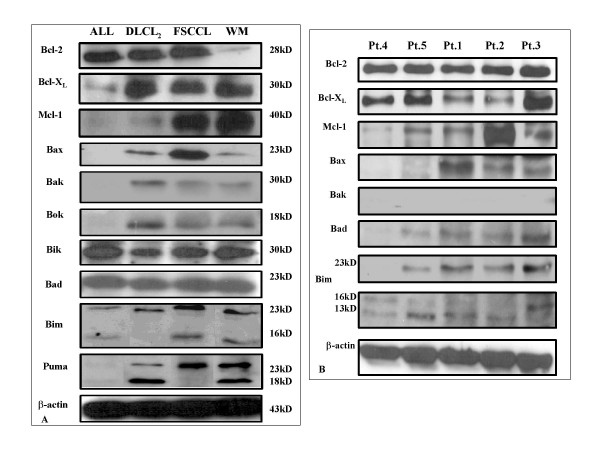
**Inventory of Bcl-2 family protein by Western-blot quantification of anti-, pro-apoptotic Bcl-2 family protein of 4 NHL cell lines (4.A) and 5 fresh patient derived samples (4.B)**. Cells were harvested and lysed for Western-blot analysis. Forty μg of total lysate was subjected to detect multi-domain anti-apoptotic proteins Bcl-2, Bcl-X_L _and Mcl-1 proteins in NHL cell lines and patient derived samples. 80 μg of total cell lysate was loaded to detect multi-domain pro-apoptotic and BH3-only Bax, Bak, Bok, Bad, Bim and Puma profiles in WSU cell lines and patient-derived fresh samples.

To determine if certain Bcl-2 family protein expression profiles are associated with increased susceptibility to TW-37, we determined the expression of major proteins in this family in all 4 cell lines and 5 of the fresh cases using Western Blotting analysis (Fig. 4). In all cases, fresh and cell lines, cells expressed at least 2 of the 3 anti-apoptotic proteins examined (Bcl-2, Bcl-X_L_, and Mcl-1). Bcl-2 was over-expressed in all fresh cases, and cell lines except the WSU-WM (expressed low levels), Bcl-X_L _was expressed in all patient cells and cell lines (except WSU-ALL cell line) and Mcl-1 was low only in WSU-ALL, WSU-DLCL2 and pt4. There was variation in the expression of the pro-apoptotic proteins examined. In every case there was at least 3 pro-apoptotic proteins expressed.

### Bcl-2 family protein of TW-37-Treated cells

**Figure 5:**
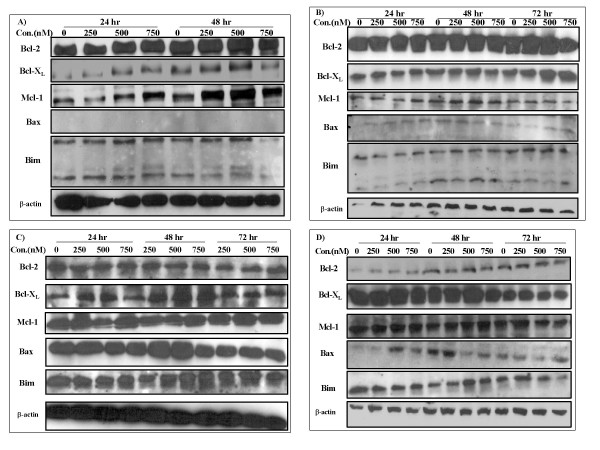
**Effect of TW-37 on the protein expression of Bcl-2, Bcl-X_L_, Mcl-1, Bax, Bim and β-actin was detected in 4 WSU cell lines, A) WSU-pre-B-ALL, B) WSU-DLCL_2_, C) WSU-FSCCL and D) WSU-WM cell lines after exposure to 250, 500 or 750 nM of TW-37 or its inactive enantiomer TW-37a for 24, 48 or 72 h, cells were harvested, lysed and analyzed by western blot analysis with indicated antibodies**.

In general Western Blot analysis conducted on all 4 cell lines exposed to different concentrations of TW-37 at various time points showed no major changes in Bcl-2 family protein levels (Fig. 5A–D). There was apparent increase of Mcl-1 in WSU-pre-B-ALL cell line at 24 and 48 hr (Fig. 5A) but similar finding was not seen in other cell lines (Fig. 5B–D). Similarly, Bcl-X_L _was more abundantly expressed in WSU-DLCL_2 _after exposure to TW-37 for 72 hr (Fig. 5B) but the finding did not extend to other cell lines. The failure of drug treatment to induce consistent change in the steady-state level of Bcl-2 family proteins implies that baseline (i.e., not drug treated) quantitation of these proteins closely approximates the quantitation in drug-treated cells, at least over the 48 to 72 hr interval.

### TW-37 blocks hetrodimerization between pro- and anti-apoptotic Bcl-2 family proteins

**Figure 6:**
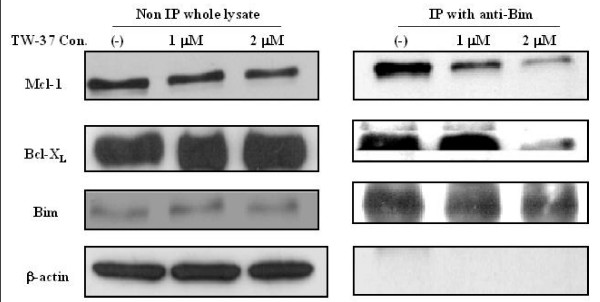
**Immunoprecipitation and western-blot analysis of heterodimerization interaction by TW-37 between anti-apoptosis and pro-apoptosis Bcl-2 family proteins**. WSU-FSCCL cells were treated with 1 or 2 μM of TW-37 for 24 hr, lysed and 300 μg of whole cell lysate was immunoprecipitated with anti-Bim followed by Western-Blot with anti-Mcl-1, anti-Bcl-X_L_, anti-Bim and anti β-actin.

Protein lysates of TW-37-treated WSU-FSCCL cells were immunoprecipitated with antibody to Bim BH3-only proapoptotic protein. Immunoprecipitates were separated by SDS-PAGE and electroblotted to a membrane. Subsequent immunoblotting with Mcl-1 and Bcl-X_L _revealed a decrease in Bim-Mcl-1 and Bim-Bcl-X_L _complexes in the WSU-FSCCL-treated cells compared with untreated (control) cell lysates (Fig. 6). The blocking of Bim-Mcl-1 heterodimerization is evident at 1 μM TW-37 and increased at 2 μM; the blocking of Bim-Bcl-X_L _heterodimerization is evident only at the highest drug concentration. This finding confirms the ability of TW-37 to block Bim-Mcl-1 and Bim-Bcl-X_L _heterodimerization. Using similar technique, previously we have shown that TW-37 blocks Bid-Bcl-2 and Bid-Mcl-1 but not Bid-Bcl-X_L _in WSU-DLCL_2 _cell lysate [27].

### *In vivo *efficacy of TW-37 in WSU-DLCL_2_-SCID mouse xenografts

The MTD of TW-37 in SCID mice was determined to be 120 mg/kg when given alone as intravenous (iv) injections (40 mg/kg daily × 3 doses). Animals at this dose experienced weight loss of < 5% and had scruffy fur, however with full recovery 48–72 hours after completion of treatment.

**Figure 7:**
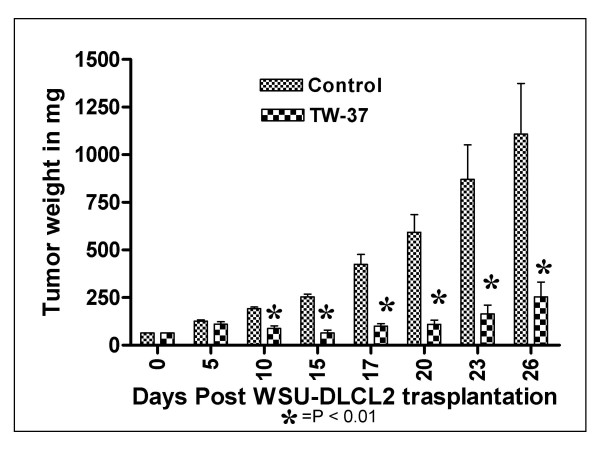
**Tumor reduction of WSU-DLCL_2 _xenograft treated with TW-37 compared with control**. TW-37 was given at 40 mg/kg by i.v. injection for three consecutive days, tumors were measured consistently; number of animals per group = 5. P value ≤ 0.01.

Antitumor activity of TW-37 at its MTD against WSU-DLCL_2_-bearing SCID mice as measured by tumor growth inhibition (T/C), tumor growth delay (T-C) and log_10_kill was 28%, 10 days and 1.5, respectively (Table 1). A T/C value of 42% or less is considered significant anti-tumor activity by NCI, the drug evaluation branch of the division of cancer treatment. Therefore, TW-37 is considered active against WSU-DLCL_2 _tumor and resulted in significant growth delay (p ≤ 0.01) compared with control (Fig. 7).

**Table 1:** Antitumor activity of TW-37 in WSU-DLCL_2 _bearing SCID mice.

Agent*	No. of Animals	T/C (%)	T-C (days)	Log_10 _Kill (Gross)
Control	5	0.0	0 22	0.0
TW-37	5	28	10	1.5

*TW-37 was given at its MTD; 40 mg/kg × 3 days, via tail vein.

## Discussion

B-cell tumors are a very heterogeneous group of diseases with diverse clinical presentations, genetic anomalies, phenotypes and natural histories [28-30]. Chemotherapy-based regimens remain the cornerstone of treating B-cell tumors but with varying results, underscoring the heterogeneity of this group of diseases despite their common B-cell lineage. It is important, therefore, that any new therapeutic strategy be evaluated across the spectrum of these tumors. This is especially important in targeted therapy of selective intracellular molecular pathways. In this study, we examined the activity of TW-37, a non-peptidic small-molecule inhibitor of pan Bcl-2 family proteins, against established human B-cell tumor lines and fresh patient samples representing the spectrum of B-cell tumors in man. Our results demonstrate activity of TW-37 across all B-cell tumors irrespective of their proliferative status, genetic abnormalities, and state of differentiation. The study also reveals the ubiquitous expression of the Bcl-2 proteins and their complexity in B-cell tumors.

Our results presented here, show that small-molecule inhibitors of the Bcl-2 family proteins has a therapeutic role in a wide spectrum of B-cell tumors. All four cell lines chosen in this study are highly proliferative, whereas the fresh patient samples have low proliferation. TW-37 was able to slow the growth of cell lines and increase the frequency of apoptotic cells in fresh patient cultures (Fig. 1). Selectivity of TW-37 toward tumor cells is demonstrated by its lack of effect on normal peripheral blood lymphocytes (Fig. 1C). Such findings indicate that the TW-37 effect, and perhaps the class it represents, is not dependent on the proliferative status of B-cell tumors. The IC_50 _of TW-37 for the cell lines ranged between 165 nM and 320 nM (Fig. 1A). In the fresh cases, the IC_50 _ranged from 300 nM to1000 nM (Fig. 1B). However, it is important to note that 1000 nM is still considered much more potent compared to most standard anticancer therapeutic drugs. It is interesting that the least sensitive (or resistant) cells (IC_50 _~1000) came from patients that were either under treatment (Pt. 6) or whose disease has progressed after treatment (Pt. 2) suggesting a possibility of cross resistance to this modality. In support of this conclusion is the observation that fresh cells from patient #6, which were obtained prior to therapy (Pt. 6A), showed more sensitivity to TW-37.

Bcl-2 was first discovered in association with the t(14;18) translocation seen in the majority of follicular lymphomas [31] and is believed to play a pivotal role in follicular lymphomagenesis. However, expression of Bcl-2 family proteins is ubiquitous in B-cell tumors and does not depend on t(14;18) or any other chromosomal translocations. All cases examined in this series including fresh samples and established cell lines expressed one or more protein in each class (Fig. 4). Over-expression or dysregulation of the Bcl-2 proteins is perhaps another common unifying theme among all B-cell tumors, which can be exploited for therapy. In this study we have demonstrated that TW-37 induces apoptosis in both patient-derived lymphoma cells and established cell lines (Fig. 2). Exposure of fresh lymphoma cells and established cell lines to TW-37 was associated with activation of caspase 3 and 9, cleavage of the polyadenosine ribose polymerase (PARP) into active fragments and DNA fragmentation (Fig. 3). These are the hallmarks of mitochondrial dependent intrinsic pathway of apoptosis [32]. Western Blot analysis conducted on all lymphoma cell lines exposed to different concentrations of TW-37 at various time points did not show dramatic decrease or increase in the anti- and pro-apoptotic proteins (Fig. 5A–D). These observations are consistent with the presumed mechanism of TW-37 action as a BH3 mimic to interfere anti- and pro-apoptotic Bcl-2 family protein interaction rather than interfere Bcl-2 family protein expression or stability and that small molecule inhibitor disrupts function but does not affect transcription of Bcl-2 family proteins. It has been suggested that the mechanism of TW-37-induced apoptosis is the blocking of heterodimerization between anti-apoptotic members, like Bcl-2, Bcl-X_L_, and Mcl-1, and pro-apoptotic members like Bax and Bak of the Bcl-2 family [33]. Our demonstration that TW-37 was able to block heterodimerization between Bim and Bcl-2 as well as Bim and Mcl-1 (Fig. 6) lends support to this mechanism.

There are other BH3-mimetic SMIs now in clinical trials, including ABT-737 [34] and GX15-070 [13]. However, TW-37 is unique in its ability to target Mcl-1 (Fig. 6). It was recently found that Mcl-1 expression is a key determinant of resistance to ABT-737 [34,35]. Mcl-1 normally acts at critical 'windows' of cell proliferation, differentiation and apoptosis [36]. Within lymphoma, Mcl-1 is expressed more abundantly in large (centroblasts) than small cells (centrocytes) [37] and its expression is associated with higher proliferation and worse prognosis [38]. In a study of the molecular mechanism of the DNA damage response during adenoviral infection, Cuconati et al. identified Mcl-1 as the key mediator [39]. Together, these studies highlight a role for Mcl-1 which was previously unrecognized. Using data from our Bcl-2 family proteins in 4 established cell lines and 7 lymphoma patients, we might be able to address some of the basic principles of the hypothesis accounting for the balance of Bcl-2 family proteins, namely, the rheostat hypothesis proposed by Korsmeyer [40-42]. The hypothesis implies that it is the *difference *between the camps (i.e., subtracting the sum of all the pro-apoptotic regulators from the sum of all the anti-apoptotic regulators). In our recent studies, we have also concluded that the Bax:Mcl-1 ratio may govern the response of lymphoma cells to BH3-mimetic small molecule inhibitors such as TW-37 [43]. The Bax:Mcl1 ratio might become a clinically-important molecular prognosticator of tumor response to TW-37 since, in this study, it correlated positively with TW-37-induced apoptosis (figure 2C).

Results of *in vivo *animals studies show that TW-37 alone is an active agent against WSU-DLCL_2 _lymphoma with tumor growth inhibition (T/C) value of 28%, tumor growth delay (T-C) of 10 days and log_10_kill of 1.50 (Table 1). Usually, a T/C value of ≤ 42% for an agent is considered active by NCI criteria. In the mouse model treatment with TW-37 resulted in statistically significant (p ≤ 0.01) delay in tumor growth when compared to control (Figure 7).

In conclusion, the use of small molecule inhibitors of pan Bcl-2 is an effective way of inducing apoptosis in a wide range of B-cell tumors in humans as well as WSU-DLCL_2 _bearing SCID mice.

## Competing interests

The authors declare that they have no competing interests.

## Authors' contributions

AMK manuscript writing, overall project direction; YS technical work; ASG data interpretation; ASA technical work; BC data interpretation; AA technical work; RMM design of research experiments, data analysis. All authors read and approved the final manuscript.
